# Implementation and Validation of Mobile Software to Promote Healthy Lifestyle in Older People: The NeoMayor Pilot Study

**DOI:** 10.1002/alz70863_110586

**Published:** 2025-12-23

**Authors:** Victoria Cabello

**Affiliations:** ^1^ Unidad de Cerebro Saludable, Hospital Clinico Universidad de Chile, Santiago, Metropolitana Chile

## Abstract

**Background:**

The accelerated aging process in Latin America increased the prevalence of cardiovascular diseases and dementia, resulting in older adults with disability. Mobile health (mHealth) applications offer opportunities for promoting healthy lifestyles in older people. In Chile there is a lack of mHealth applications with multimodal lifestyle interventions, and evidence supports their effectiveness. Our aim is to describe the development and validation of the mHealth NeoMayor.

**Method:**

I. Design & Development: we conducted 26 co‐creation sessions with users, consulted experts, and made a literature review and worked with a team of app development to create a functional prototype of NeoMayor app. II. Validation: we performed a pilot study with 60 independent and cognitively normal subjects. Clinical and cognitive assessments were applied before and after the two months use of NeoMayor app.

**Result:**

Participants showed significant cardiovascular health improvements, a mean increase in the Life´s Essential 8 cardiovascular health index score from 64 ±10 to 68 ± 11 (*p* <0.001), significant reductions in systolic blood pressure (10 mmHg), waist circumference (6 cm), weight and fasting glucose levels. Also, improvement in International Physical Activity Questionnaire score from 1,8 ± 0,6 to 2,4 ± 0,6, in the Short Physical Performance Battery from 11.3 ± 1 to 11.9 ± 0.5; and the Timed Up and Go test from 10±2 to 8±2 seconds, and in Mediterranean‐DASH Diet Intervention for Neurodegenerative Delay food score from 1.8 ± 0.6 to 2.4 ± 0.6.

**Conclusion:**

Is the first initiative in Chile to develop and validate a mHealth tool to promote healthy lifestyles, cardiovascular and brain health in older adults with significant improvements. NeoMayor app offers an effective, accessible, and affordable solution for promoting healthy aging.

